# Comprehensive Profile of Pathogens and Antimicrobial Resistance in Conjunctivitis Cases from Niger

**DOI:** 10.4269/ajtmh.23-0498

**Published:** 2023-11-06

**Authors:** Abdou Amza, Beido Nassirou, Boubacar Kadri, Saley Ali, Boubacar Mariama, Cissé Mamadou Ibrahim, Lamine Aboubacar Roufaye, Elodie Lebas, Emily Colby, Lina Zhong, Cindi Chen, Kevin Ruder, Danny Yu, YuHeng Liu, Thomas Abraham, Aaron Chang, Lina Mai, Armin Hinterwirth, Gerami D. Seitzman, Thomas M. Lietman, Thuy Doan

**Affiliations:** ^1^ Programme Nationale de Santé Oculaire, Niamey, Niger;; ^2^ Francis I. Proctor Foundation, University of California, San Francisco, California;; ^3^ Department of Ophthalmology, University of California, San Francisco, California;; ^4^ Department of Epidemiology and Biostatistics, University of California, San Francisco, California;; ^5^ Institute for Global Health Sciences, University of California, San Francisco, California

## Abstract

Infectious conjunctivitis outbreaks remain a public health burden. This study focuses on the pathogen and antimicrobial resistance (AMR) profiles identified in Niger. Sixty-two patients with acute infectious conjunctivitis who presented to health posts were enrolled from December 2021 to May 2022. Nasal and conjunctival swabs were obtained from each patient. Unbiased RNA deep sequencing (RNA-seq) was used to identify associated pathogens. A pathogen was identified in 39 patients (63%; 95% CI, 50–74). Of those, an RNA virus was detected in 23 patients (59%; 95% CI, 43–73). RNA viruses were diverse and included human coronaviruses (HCoVs): SARS-CoV-2, HCoV-229E, HCoV-HKU1, and HCoV-OC43. A DNA virus was identified in 11 patients (28%; 95% CI, 17–44). Of those, four patients had a coinfection with an RNA virus and two patients had a coinfection with both an RNA virus and a bacterium. DNA viruses were predominantly human herpesvirus (cytomegalovirus, Epstein–Barr virus, human herpesvirus 8) and human adenovirus species B, C, and F. Eighteen patients (46%; 95% CI, 32–61) had a bacteria-associated infection that included *Haemophilus influenza*, *Haemophilus aegyptius*, *Staphylococcus aureus*, *Streptococcus pneumoniae*, and *Moraxella* spp. Antimicrobial resistance determinants were detected in either the conjunctiva or nasal samples of 20 patients (32%; 95% CI, 22–45) and were found to be more diverse in the nose (Shannon alpha diversity, 1.12 [95% CI, 1.05–1.26] versus 1.02 [95% CI, 1.00–1.05], *P* = 0.01). These results suggest the potential utility of leveraging RNA-seq to surveil pathogens and AMR for ocular infections.

## INTRODUCTION

Conjunctivitis is a feature of many infectious disease outbreaks, and recognition of the ocular signs and symptoms is critical in minimizing the spread of the disease.^1^^,^^2^ In the United States and the developing world, outpatient conjunctivitis is thought to be predominantly viral in etiology.^3^ In this setting, the appropriate clinical management is hand hygiene, limitation of social contacts, and conservative care without antimicrobials. In other parts of the world, such as Burkina Faso, a sub-Saharan African country, the pathogen profile is more diverse and, in some cases, antimicrobials may be appropriate and even lifesaving.^4^ Thus, the general call to curb antibacterial treatment of acute infectious conjunctivitis to minimize antimicrobial resistance (AMR) may need to be reinterpreted within both social and geographic contexts.^3^^,^^5^^,^^6^

Niger is a low- to middle-income country in West Africa that borders Burkina Faso. It has a high burden of morbidity and mortality, and mass drug distributions for infectious diseases are common.^7^^–^^9^ The country has a health system to provide some essential health care, including conjunctivitis. However, because accessibility to laboratory testing is limited, the microbial characterization of pathogens associated with acute conjunctivitis is sparse for Niger. In this context, we aimed to determine 1) the signs and symptoms, and 2) the pathogen profile associated with acute infectious conjunctivitis. Furthermore, we characterized the existing AMR determinants, or resistome, in patients at the individual level, demonstrating the potential utility of pathogen detection and concurrent passive AMR surveillance.

## MATERIALS AND METHODS

### Study design and setting.

Study inclusion criteria included symptoms of presumed infectious conjunctivitis within 14 days. Exclusion criteria included presumed allergic, medication-related conjunctivitis or refusal to provide consent. All participating health-care personnel were trained in the swabbing protocol prior to study initiation. All patients presenting to the outpatient ophthalmology service at Niamey National Hospital who met the study criteria were enrolled, examined, and underwent sample collection during the same visit. Ethical approval from the institutional review board of the University of California, San Francisco and the ethical committee of the Niger Ministry of Health was obtained. This study adhered to the tenets of the Declaration of Helsinki. Written consent from participants 18 years and older was obtained. Guardians provided consent for children younger than 18 years of age. No incentives were offered for participating in this study.

### Sample collection and management.

Sterile polyester applicators (Puritan) were used to obtain three swabs (two conjunctival swabs and one swab for both anterior nares). The swabs were then stored in DNA/RNA-Shield (Zymo Research, Irvine, CA) and transferred to a –80°C freezer until shipment back to the University of California, San Francisco for long-term storage until sample processing.^4^^,^^10^

### Sample processing and bioinformatics.

All samples were de-identified and processed in a random manner. RNA deep sequencing (RNA-seq) library preparation, sequencing, and bioinformatics analyses for pathogen detection and AMR have been described previously.^10^ Briefly, 5 µL of extracted RNA from each sample was converted to complementary DNA, and sequencing libraries were prepared using the NEBNext ULTRA II RNA Library Prep Kit for Illumina (New England Biolabs, Ipswich, MA). The samples were sequenced using a NovaSeq 6000 system (Illumina, San Diego, CA) with 150-nucleotide paired-end sequencing.

Host reads were removed, with the remaining nonhost reads filtered for quality. Those nonhost reads passing the quality filter were aligned 1) to an entire National Center for Biotechnology Information nonredundant collection database for pathogen detection and 2) to the MEGARes reference antimicrobial database (version 1.0.1), using the Burrows-Wheeler Aligner with default settings.^11^ As bacteria colonize the nares, only viruses were considered for nasal samples to minimize overinterpretation. Matched antimicrobial genetic resistance determinants were grouped at the class level and subjected to further statistical analyses.^12^ Shannon alpha diversities were compared by permutation testing using R (version 3.5; R Foundation for Statistical Computing, Vienna, Austria).^13^ Ninety-five percent CIs (95% CI) were acquired using the adjusted Wald method. Figures were generated using Prism (version 10.0.3; GraphPad Software, Boston, MA).

## RESULTS

Table 1 summarizes the demographics of the 62 patients enrolled in the 6-month period from December 2021 to May 2022. Of these patients, 33 were female (57%). The mean age was 24 ± 21 years (SD) and a mean onset of symptoms of 5 ± 3 days. Documented ocular symptoms included purulent discharge (60%; 95% CI, 47–71), itching (40%; 95% CI, 29–53), and tearing (30%; 95% CI, 19–42). On examination, bilateral subepithelial infiltrates were noted in 80% of patients (95% CI, 64–85). Coughing was the most common systemic finding (23%; 95% CI, 14–35), followed by rhinorrhea (16%; 95% CI, 9–27), and sore throat (7%; 95% CI, 2–16). Twenty-one percent of patients (95% CI, 11–36) reported having personal contact with individuals with similar signs and symptoms.

**Table 1:** Demographics and clinical signs and symptoms of enrolled patients

Demographic	Clinical sign	*n* *	%	95% CI†
Sex‡				
Female	–	33	57	–
Male	–	25	43	–
Age, years ± SD	–	24 ± 21	–	–
Duration of symptoms, days	–	5 ± 3	–	–
Ocular symptoms	Purulent discharge	37/62	60	47–71
	Itching	25/62	40	29–53
	Tearing	18/61	30	19–42
Examination findings	Bilateral	47/62	80	64–85
	Subepithelial infiltrates	0/60	0	0–5
	Membranes	0/61	0	0–5
	Preauricular lymphadenopathy	0/59	0	0–5
Systemic findings	Sore throat	4/59	7	2–16
	Runny nose	10/62	16	9–27
	Cough	14/61	23	14–35
Contacts similarly affected	Yes	9/42	21	11–36
Presented on antibiotic drop§	Yes	9/62	13	8–26

* Or *n*/*N*.

† Adjusted Wald method.

‡ Sex known for 58 of 62 participants.

§ Antibiotic eyedrops known at presentation. An additional seven patients were on eyedrops of unknown name.

**Figure 1.**
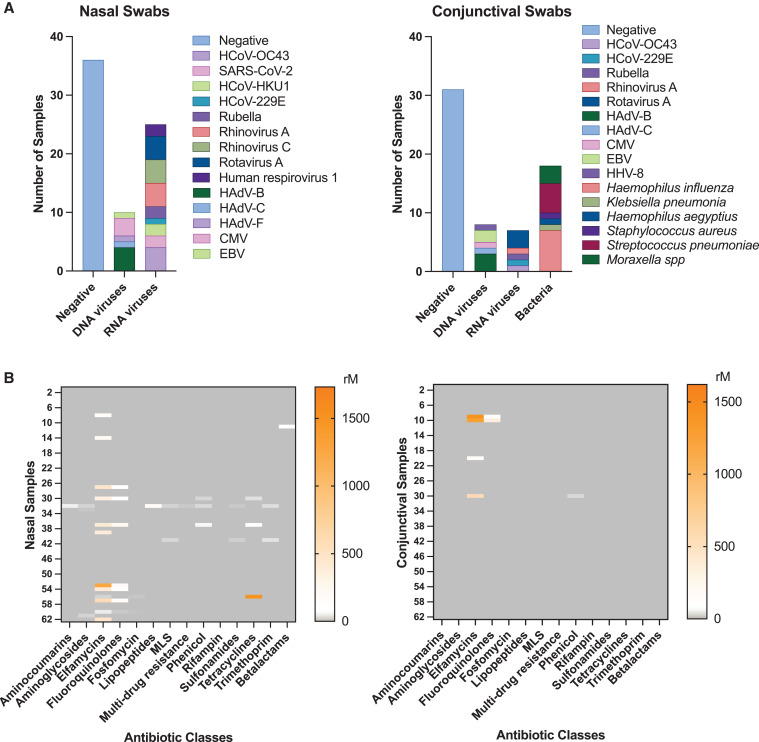
Summary of pathogen profiles and genetic resistance determinants for nasal and conjunctival samples. (**A**) Stacked bar graph of pathogens identified for nasal samples (left) and conjunctival samples (right). (**B**) Heatmaps of normalized reads of genetic resistance determinants for each antibiotic class. Each row represents the reads per million matched reads (rM) for the nasal sample (left panel) and a conjunctival sample of a patient (right panel). CMV = cytomegalovirus; EBV = Epstein–Barr virus; HAdV = human adenovirus; HCoV = human coronavirus; HHV = human herpesvirus; MLS = macrolides, lincosamides, and streptogramins.

A combined 186 samples (62 nasal and 124 conjunctiva) were processed for RNA-seq. An associated pathogen was identified in 39 patients (63%; 95% CI, 50–74) and could be grouped into three categories: bacteria, DNA viruses, and RNA viruses (Figure 1A). Of those that were positive, RNA pathogens predominated and were detected in 59% of patients (23 of 39 patients; 95% CI, 43–73). Human coronaviruses (HCoVs) were detected in nine patients and included both the alpha (HCoV-229E) and beta (HCoV-OC43, HCoV-HKU1, and SARS-CoV-2) genera. Of the two patients with SARS-CoV-2–associated conjunctivitis, one 33-year-old woman presented with unilateral eye involvement with respiratory involvement (coughing and sore throat), whereas the other patient was a 13-year-old girl with bilateral eye involvement and no respiratory symptoms. Other viruses included rubella, rhinovirus A and C, rotavirus A, and human respirovirus 1.

The majority of the DNA viruses fell into two different pathogen types: human adenovirus (HAdV) and human herpesvirus (HHV). Unlike the United States and India, where HAdV species D is commonly associated with acute conjunctivitis, HAdV-B, -C, and -F were detected in our Nigerien patient population. Of the human herpesviruses, cytomegalovirus, Epstein–Barr virus, and HHV-8 (Kaposi sarcoma herpesvirus) were detected. Human herpesvirus 8 was detected on the conjunctiva of a 73-year-old woman who presented with a 10-day onset of bilateral conjunctivitis. She also reported having a sore throat, rhinorrhea, and coughing. Her nasal sample was positive for rotavirus A. Of the 25 patients with at least one virus identified in the nose (either DNA virus or RNA virus), 36% (95% CI, 20–55) had the same virus identified in their conjunctival samples.

Bacterial infection was identified on the conjunctiva of 18 patients (46%; 95% CI, 32–61) and included *Haemophilus influenza*, *Haemophilus aegyptius*, *Staphylococcus aureus*, *Streptococcus pneumoniae*, and *Moraxella* spp. Coinfections were documented in 14 of 39 patients (36%; 95% CI, 23–52), with both bacteria and virus identified in seven patients (18%; 95% CI, 9–32). In addition, four patients with coinfections of DNA and RNA viruses, two patients with multiple RNA viruses, and one patient with a coinfection of different DNA viruses were noted.

Figure 1B summarizes the genetic resistant determinants isolated from nasal and conjunctival swabs. Overall, normalized reads of genetic determinants of resistance to different antibiotic classes varied across each patient and were detected most commonly in the elfamycin, fluconazole, and tetracycline classes. The prevalence of at least one class of genetic resistance determinants was greater in nasal samples compared with conjunctival samples (27% [95% CI, 18–40] versus 6% [95% CI, 3–15], *P* = 0.003). Diversity of genetic determinants was also greater for nasal samples compared with conjunctival samples (Shannon alpha diversity, 1.12 [95% CI, 1.05–1.26] versus 1.02 [95% CI, 1.00–1.05], *P* = 0.01). Of the nine patients who presented on antibiotic eyedrops, none was found to have detectable AMR determinants in their conjunctival samples. However, the duration of antibiotic use was not documented as part of the clinical assessment.

## DISCUSSION

Pathogens associated with conjunctivitis outbreaks are increasingly recognized as being diverse and varying geographically. The advantage of unbiased RNA-seq is the ability to identify any pathogen (bacteria, fungi, parasites, DNA and RNA viruses) in a clinical sample. Here, we used this technology with the aim of monitoring pathogens in patients with acute conjunctivitis while tracking AMR concurrently in the same samples. Overall, we found that viral and bacterial etiologies were common. We also found that the anterior nares and ocular surface are potentially easy-access, less-invasive sites for genomic surveillance of AMR at the individual level in a population undergoing routine mass drug distributions.

Human coronaviruses are a group of enveloped, positive-sense, single-stranded RNA viruses. To date, seven HCoVs have been identified, and they can be divided into two genera: alphacoronavirus and betacoronavirus.^14^ Of these HCoVs, HCoV-229E, HCoV-NL63, HCoV-HKU1, and HCoV-OC43 generally cause mild respiratory symptoms and conjunctivitis.^15^^,^^16^ SARS-CoV, SARS-CoV-2, and Middle East respiratory syndrome-CoV, on the other hand, can cause severe respiratory symptoms.^14^ Recently, work from the Seasonal Conjunctivitis Outbreak Reporting for Prevention and Improved Outcomes (SCORPIO) study group and others^10^^,^^17^^–^^19^ have shown that SARS-CoV-2 genomic material can be isolated directly from the conjunctiva of patients with acute conjunctivitis who presented to outpatient clinics. This demonstrates that SARS-CoV-2 can be associated with primary conjunctivitis even without associated respiratory symptoms. In our Nigerien cohort, the common human circulating coronaviruses (HCoV-229E, HCoV-HKU1, and HCoV-OC43) were detected in seven patients. Five patients had bilateral eye involvement and three patients reported respiratory symptoms (coughing, sore throat, or rhinorrhea). Of the two patients with SARS-CoV-2–associated conjunctivitis, only one patient reported respiratory symptoms. This suggests that HCoV may be an important and underappreciated pathogen class for outpatient conjunctivitis in Niger.

We identified two children, 4 and 5 years old, with rubella-associated conjunctivitis. Both children had bilateral eye involvement and coughing as a respiratory complaint. There was no evidence of corneal involvement on examination, although symptom onset at enrollment was only 3 days. Rubella is a positive-sense, single-stranded RNA virus.^20^^,^^21^ It is one of the leading causes of preventable birth defects and is transmitted via airborne droplets.^21^ Infection during the gestational period can result in birth defects, hearing loss, and blindness. The disease is mild, however, and can occur in children and adults, with the common clinical manifestations of mild fever, transient body rash, and conjunctivitis. In some patients, this initial infection can result in an indolent form of intraocular inflammation because the virus can seed intraocularly and remain in the eye—an immune-privileged site—for quite some time.^22^ Rubella vaccination is routine in many countries, although it is currently not a component of the childhood vaccination program in Niger.^23^

Human adenoviruses are nonenveloped, double-stranded DNA viruses and are one of the most frequent etiological agents for respiratory tract infections and acute viral conjunctivitis worldwide.^24^^,^^25^ Human adenoviruses are classified into seven species (A–G) that are subdivided further into genotypes. Conjunctivitis outbreaks are generally associated with various HAdV-D genotypes, although ocular involvement resulting from genotypes HAdV-B, HAdV-C, and HAdV-E have been detected.^26^ In our study, we detected multiple species (B, C, and F) associated with acute conjunctivitis. This HAdV profile appears more diverse than in neighboring Burkina Faso, although the differences may also be attributed to seasonality or small sample size.^4^

Various bacteria-associated etiologies of infectious conjunctivitis were also identified. Both gram-negative (*H. influenza*, *H. aegyptius*, *Klebsiella pneumoniae*, and *Moraxell*a spp.) and gram-positive (*S. aureus* and *S. pneumoniae*) bacteria were detected. *Haemophilus aegyptius* was found to be the causative pathogen in a 3-year-old boy who presented with bilateral purulent conjunctivitis. *Haemophilus aegyptius* was first identified by Koch^27^ in Egyptian patients with conjunctivitis and was known to cause outbreaks. In the 1980s, *H. aegyptius* was also associated with Brazilian purpuric fever epidemics during which infected children presented with purulent conjunctivitis that progressed to systemic symptoms including fever, gastrointestinal distress, and purpura fulminans.^28^ Disease progression is an otherwise previously healthy child is rapid, and death can occur.^28^ As SCORPIO is a disease surveillance study, and follow-up visits are not included in the study design, it is unknown whether the child with *H. aegyptius* detected in this study developed systemic sequalae.

Patients with infectious conjunctivitis secondary to bacterial etiologies may benefit from antibiotics or close observation, particularly in children. However, from an antibiotic stewardship standpoint, there is a concern that mass antibiotic distribution may facilitate the spread of antibiotic resistance. Because viral etiology is thought to be predominant in cases of conjunctivitis in the United States, indiscriminate dispensing of antibiotic eyedrops for conjunctivitis in the United States may be inappropriate.^3^^,^^29^^,^^30^ However, testing for pathogen identification is not performed routinely for conjunctivitis in any location. Thus, targeted treatment toward definitively identified pathogens is rarely possible. Understanding the baseline prevalence of pathogens and of AMR profiles in these patients may improve decision workflow for patients with infectious conjunctivitis in Niger. In addition, with seasonal malaria chemoprevention during the rainy season in Niger and with the documented increase in consumption of fluoroquinolones and beta-lactams in the region, there is additional motivation to quantify local pathogen resistance patterns.^31^^–^^33^ In our study, we found the prevalence of AMR determinants was greater in the anterior nares than in the conjunctiva. In addition, the nasal resistome was more diverse than the ocular surface resistome. One nasal sample from a 15-day-old baby boy with *Moraxella catarrhalis*–associated bilateral conjunctivitis harbored genetic resistance from eight classes of antibiotics (aminocoumarins, aminoglycosides, lipopeptides, macrolides, multidrug resistance, phenicol, sulfonamides, and trimethoprim). The patient did not present on antibiotic eyedrops. Thus, it may be that he was exposed to antibiotics during the gestational period or shortly thereafter. These results suggest the anterior nares may be a minimally invasive site for the detection of AMR.

The strengths of our study include the use of unbiased RNA-seq to survey comprehensively all potential pathogens associated with conjunctivitis in Niger, where phenotypic testing remains a challenge because pathogen viability for culturing purposes is hard to maintain during transportation. In addition, we were able to provide concurrent information on AMR at the individual level. Limitations of our surveillance study include a small sample size and the lack of longitudinal data to evaluate clinical outcomes. These limitations may contribute to the lack of preauricular lymphadenopathy and conjunctival membranes or pseudomembranes documented in our patient cohort. Although the detection of genetic material does not always correlate definitively with causation, clinical testing has, overall, become more reliant on molecular diagnostics such as nucleic acid amplification tests. Careful clinical correlation will always be important in the determination of causation. In particular, associated viruses detected in nasal swabs may be indicative of colonizers, and not causative pathogens, in some patients. Furthermore, it is unclear whether the resistome identified had direct phenotypic or clinical correlations. The low-biomass nature of conjunctival samples may limit the sensitivity of RNA-seq and may explain the less diverse resistome compared with the resistome of the anterior nares. Lastly, the pathogen profile as described in our study should only be interpreted in the context of Niger, because it has become increasingly clear that geography is an important factor in understanding infectious conjunctivitis.^34^

In summary, this study revealed that pathogens associated with acute conjunctivitis in Niger are heterogenous. Because comprehensive, unbiased pathogen-directed testing is currently impractical on an individual basis, periodic comprehensive community surveillance may be helpful in the identification of changes in disease patterns and of emerging infectious diseases.

## Financial Disclosure

The research reported herein was supported by the National Eye Institute of the NIH (Award No. R01EY032041) and by a Research to Prevent Blindness unrestricted grant.
